# A CRE/DRE dual recombinase transgenic mouse reveals synaptic zinc–mediated thalamocortical neuromodulation

**DOI:** 10.1126/sciadv.adf3525

**Published:** 2023-06-09

**Authors:** Stylianos Kouvaros, Brandon Bizup, Oscar Solis, Manoj Kumar, Emilya Ventriglia, Fallon P. Curry, Michael Michaelides, Thanos Tzounopoulos

**Affiliations:** ^1^ Pittsburgh Hearing Research Center, Department of Otolaryngology, University of Pittsburgh, Pittsburgh, PA 15261, USA.; ^2^ Biobehavioral Imaging and Molecular Neuropsychopharmacology Unit, National Institute on Drug Abuse Intramural Research Program, Baltimore, MD 21224, USA.; ^3^ Department of Psychiatry and Behavioral Sciences, Johns Hopkins University School of Medicine, Baltimore, MD 21205, USA.

## Abstract

Synaptic zinc is a neuromodulator that shapes synaptic transmission and sensory processing. The maintenance of synaptic zinc is dependent on the vesicular zinc transporter, ZnT3. Hence, the ZnT3 knockout mouse has been a key tool for studying the mechanisms and functions of synaptic zinc. However, the use of this constitutive knockout mouse has notable limitations, including developmental, compensatory, and brain and cell type specificity issues. To overcome these limitations, we developed and characterized a dual recombinase transgenic mouse, which combines the Cre and Dre recombinase systems. This mouse allows for tamoxifen-inducible Cre-dependent expression of exogenous genes or knockout of floxed genes in ZnT3-expressing neurons and DreO-dependent region and cell type–specific conditional ZnT3 knockout in adult mice. Using this system, we reveal a neuromodulatory mechanism whereby zinc release from thalamic neurons modulates *N*-methyl-d-aspartate receptor activity in layer 5 pyramidal tract neurons, unmasking previously unknown features of cortical neuromodulation.

## Teaser

Synaptic zinc from thalamic neurons modulates NMDA receptor activity in cortical neurons.

## INTRODUCTION

Zinc is essential to life. As a constituent of nearly 3000 mammalian proteins, zinc plays a key role in protein structure, enzymatic catalysis, and cellular regulation (*1*). Although the chemistry and biology of zinc metalloproteins have historically dominated the field of zinc biology, there is a growing appreciation for a signaling role of mobile zinc found in secretory tissues such as the prostate, pancreas, and, especially, the brain (*2*). In the brain, it has been known for more than two decades that the vesicular zinc transporter ZnT3 sequesters zinc into the synaptic vesicles of a substantial subset of neurons (*3*–*5*). This discovery has generated the fascinating hypothesis that vesicular, synaptically released (synaptic) zinc is a neuromodulator in glutamatergic and γ-aminobutyric acid (GABA)–releasing (GABAergic) synapses (*3*, *6*, *7*). During the past 10 years, studies from our laboratories and others have established that synaptic zinc inhibits GluN2A *N*-methyl-d-aspartate receptors (NMDAR) and AMPA receptors (AMPAR), potentiates GABA receptors, and modulates sensory processing (*3*, *5*, *8*–*19*). In the auditory cortex (AC), synaptic zinc signaling shapes sound processing by modulating the response gain and frequency tuning of layer 2/3 (L2/3) principal neurons (PNs) and somatostatin (SOM) and parvalbumin (PV) interneurons in a cell type–specific manner, and this modulation is associated with improved sound frequency discrimination (*13*, *14*). Moreover, synaptic zinc signaling is necessary for cortical adaptation to changes in background sound stimulus statistics (*20*). Thus, synaptic zinc fine-tunes neurotransmission and sensory processing.

The pattern of *Znt3* mRNA expression is consistent with the pattern of histochemically stained vesicular zinc (*21*), and this stainable zinc is absent in ZnT3 knockout (KO) mice (*4*). This finding has rendered the ZnT3 KO mouse a key tool for studying the mechanisms and roles of synaptic zinc. Many previous studies have described numerous deficits associated with ZnT3 KO mice (*7*, *19*, *22*–*25*), but given the constitutive lack of ZnT3, it is possible that some of the observed phenotypes might be due to developmental deficits and not directly to the actions of ZnT3 and vesicular zinc in adult mice. In addition, there are numerous studies where these KO mice do not show any deficits in physiological or behavioral assays that are associated with zincergic synapses (*7*). It is possible that potential roles of zinc signaling might be obscured by compensatory mechanisms due to the lack of ZnT3 throughout development. Moreover, the identification of zincergic neurons is limited by the existing histochemical technique, which does not label all known zincergic cell bodies, as it relies on the retrograde transport of zinc selenide from the axon terminal to the cell body of origin (*26*, *27*). Last, with the current tools and techniques, it is not feasible to silence or stimulate zincergic neurons in either a cell type–specific or anatomically specific manner to examine their functional role.

**Fig. 1.**
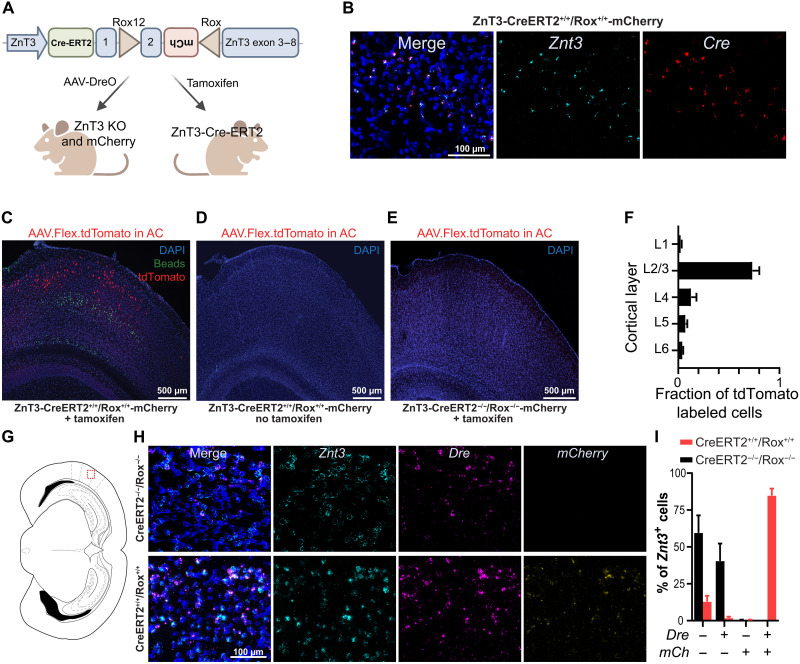
Genetic construct design and validation of ZnT3-CreERT2/Rox-mCherry mouse. (**A**) Schematic illustration showing the genetic strategy of ZnT3-CreERT2/Rox-mCherry mouse. (**B**) Representative histology of *Znt3* and *Cre* mRNA expression in AC L2/3 from a transgenic ZnT3-CreERT2^+/+^/Rox^+/+^-mCherry mouse. (**C**) Stitched image of the AC of a ZnT3-CreERT2^+/+^/Rox^+/+^-mCherry mouse injected with AAV.Flex.tdTomato and treated with tamoxifen for 5 days. Putative *ZnT3*-expressing cells express tdTomato (red). L5 PT PNs labeled via green retrobeads injection in the inferior colliculus (IC). 4′,6-Diamidino-2-phenylindole (DAPI) labels the nuclei of all cells (blue). (**D**) ZnT3-CreERT2^+/+^/Rox^+/+^-mCherry mouse injected with AAV.Flex.tdTomato but not treated with tamoxifen. (**E**) ZnT3-CreERT2^−/−^/Rox^−/−^-mCherry mouse injected with AAV.Flex.tdTomato and treated with tamoxifen for 5 days to induce CRE expression. (**F**) Distribution of tdTomato labeled (ZnT3-expressing) neurons across all cortical layers (means ± SEM; *n* = 5 mice, five slices per mouse). (**G** to **I**) Comparison of *Znt3*, *Dre*, and *mCherry* expression mRNA in mouse AC L2/3. (G) Coronal scheme depicting the region analyzed (red square). (H) Confocal images showing representative images of RNAscope FISH of *Znt3* mRNA (cyan), *Dre* mRNA (magenta) and *mCherry* mRNA (yellow) expression in the AC of ZnT3-CreERT2^−/−^/Rox^−/−^-mCherry (top) and ZnT3-CreERT2^+/+^/Rox^+/+^-mCherry (bottom) mice. (I) Quantification of the proportion of *Znt3* cells that colocalize with *Dre* and/or *mCherry* (*mCh*) [means ± SEM; *n* = 2 wild-type (WT) and 3 ZnT3-CreERT2^+/+^/Rox^+/+^-mCherry mice, *n* = 2 sections per mouse].

To overcome these limitations, we developed a new transgenic/knock-in mouse, the dual recombinase ZnT3-CreERT2/Rox-mCherry mouse, which allows for the concomitant use of Cre-Lox and DreO-Rox recombinase systems in vivo. These mice express a tamoxifen-inducible CRE recombinase estrogen receptor mutant (ERT2) fusion protein in ZnT3-expressing cells and contain Rox recombination sites flanking the ZnT3 exon 2 locus and an inverted mCherry reporter sequence (Fig. 1A). This dual recombinase transgenic mouse, which combines the Cre and Dre recombinase systems (Fig. 1A), allows for Cre-dependent expression of exogenous genes or KO of floxed genes in ZnT3-expressing cells and Dre-dependent conditional KO of ZnT3 (coupled with mCherry reporter expression; Fig. 1A) in adult mice (*28*). Thus, the Cre system allows for the transgenic expression of genetically encoded constructs to selectively label and genetically manipulate targeted populations of zincergic neurons. Furthermore, targeted expression of the DreO recombinase via viral vectors, or crossing of these mice with DreO-expressing lines, can generate region- and cell type–specific conditional ZnT3 KO in adult mice (*28*). Here, we use this unique transgenic mouse to establish previously unknown biological insights into thalamocortical neuromodulation.

## RESULTS

### Generation and validation of the ZnT3-CreERT2/Rox-mCherry transgenic mouse

ZnT3-CreERT2^+/+^/Rox^+/+^-mCherry mice were generated on a C57BL/6 background. The 5′ CreERT2 was in-frame fused to the endogenous ATG start site of the mouse *Slc30a3* gene (*Znt3*), and the 3′ CreERT2 was fused with the P2A–exon 1 coding sequence to allow for expression of CreERT2 and ZnT3 (Fig. 1A and fig. S1). The flippase recognition target (FRT)–flanked neomycin selection cassette was inserted between these two sequences, and upon deletion of this cassette, the CreERT2 sequence was spliced to become functional. The inverted mCherry cassette was inserted in intron 2. Exon 2 of ZnT3 and the inverted mCherry cassette were flanked by ROX12 and ROX sites for DreO-mediated recombination and conditional expression of the mCherry reporter (Fig. 1A).

To validate the line, we first used RNAscope fluorescent in situ hybridization (FISH) to confirm expression of *Cre* mRNA, intact expression of *Znt3* mRNA, and colocalization of *Cre* mRNA with *Znt3* mRNA. We focused on AC L2/3 , where previous studies have revealed abundant zincergic cells (*27*), and found that *Cre* mRNA was detected only in *Znt3*-positive cells (Fig. 1B). In contrast, no *Cre* mRNA was observed in *Znt3*-negative cells (Fig. 1B), confirming that ZnT3-CreERT2^+/+^/Rox^+/+^-mCherry mice express CRE recombinase specifically in *Znt3*-positive cells.

Next, we tested the ability of the expressed Cre-ERT2 to induce selective and tamoxifen-induced recombination in *Znt3*-positive cells in the AC. To demarcate the AC boundaries, we injected ZnT3-CreERT2^+/+^/Rox^+/+^-mCherry mice with green fluorescent retrograde beads into the inferior colliculus (IC), so that green fluorescent–labeled AC cells would correspond to L5 corticocollicular neurons [CCols; also termed pyramidal tract (PT)] (Fig. 1C) (*29*). We also injected the AC of these mice with a Cre-dependent adeno-associated viral (AAV) vector (AAV.Flex.tdTomato), followed by treatment with tamoxifen for 5 days. Four weeks after the injection, we examined mice for tdTomato expression (red) and retrobead fluorescence (green) in the AC (Fig. 1C). Consistent with previous reports for ZnT3 expression (*27*), the majority of ZnT3-positive tdTomato-labeled neurons were localized to L2/3 (Fig. 1F). We did not observe any tdTomato-positive cells in ZnT3-CreERT2^+/+^/Rox^+/+^-mCherry mice that were injected with AAV.Flex.tdTomato but not injected with tamoxifen (Fig. 1D) or in ZnT3-CreERT2^−/−^/Rox^−/−^-mCherry mice injected with AAV.Flex.tdTomato and treated with tamoxifen (Fig. 1E). These results validate the selectivity of the transgenic mouse to ZnT3- expressing cells, only in the presence of tamoxifen.

Subsequently, to validate the efficacy of DreO/Rox-mediated recombination at the ZnT3 locus, we first injected AAV.Syn.DreO in ZnT3-CreERT2^+/+^/Rox^+/+^-mCherry and ZnT3- CreERT2^−/−^/Rox^−/−^-mCherry mice into the AC and then performed RNAscope FISH experiments. ZnT3-CreERT2^+/+^/ Rox^+/+^-mCherry and ZnT3-CreERT2^−/−^/Rox^−/−^-mCherry mice showed similar *Znt3* and *DreO* expression (Fig. 1H). This was expected because the AAV.Syn.DreO transduces all neurons and the available *Znt3* RNAscope probe does not distinguish cells with intact versus excised *Znt3*. CreERT2^+/+^/Rox^+/+^-mCherry mice expressed *mCherry* only when *DreO* was coexpressed (Fig. 1, H and I). In ZnT3-CreERT2^+/+^/Rox^+/+^-mCherry mice, ~85% of *Znt3*-expressing cells also coexpressed *DreO* and *mCherry* (Fig. 1, H and I). ZnT3-CreERT2^−/−^/Rox^−/−^-mCherry did not express mCherry either in the presence or in the absence of *DreO* expression (Fig. 1, H and I). Together, these results validate the specificity and indicate the efficacy of DreO/Rox-mediated recombination at the ZnT3 locus.

To validate whether these mice can induce cell type– specific DreO-mediated recombination, we crossed ZnT3- CreERT2^+/+^/Rox^+/+^-mCherry mice with PV-Dre^+/+^ mice (*28*), which express DreO in PV neurons. Consistent with the genetic strategy shown in Fig. 1A, expression of mCherry (after DreO- mediated recombination) is found only in ZnT3-Cre^+/−^/ Rox^+/−^::PV-Dre^+/−^ mice and only in PV-positive interneurons (fig. S2), indicating cell type–specific DreO-mediated recombination.

**Fig. 2.**
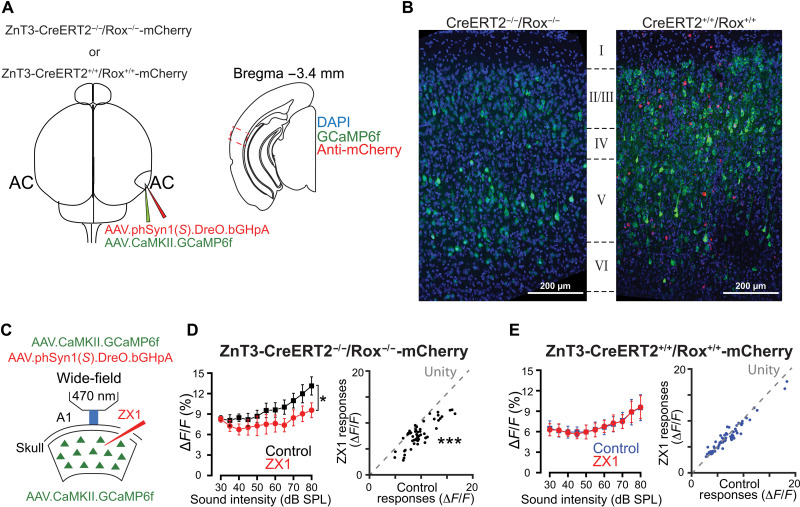
Validation of DreO-mediated of functional deletion of ZnT3. (**A** and **B**) Virally-mediated *Znt3* KO in AC pyramidal neurons. (A) Left: Schematic illustration of stereotaxic injections of two viral vectors (AAVs); one for GCaMP6f expression (AAV.CaMKII.GCaMP6f, green) and one for ZnT3 ablation [AAV.phSyn1(*S*).DreO.bGHpA] in AC neurons in ZnT3-CreERT2^+/+^/Rox^+/+^-mCherry or ZnT3-CreERT2^−/−^/Rox^−/−^-mCherry mice. Right: Illustration of AC slice showing the imaging area. (B) Stitched images of AC of injected ZnT3-CreERT2^−/−^/Rox^−/−^-mCherry (left) and ZnT3-CreERT2^+/+^/Rox^+/+^-mCherry (right) mice. mCherry expression (red) indicates ZnT3-KO cells. (**C**) Schematic of the experimental setup illustrating in vivo transcranial wide-field GCaMP6f calcium imaging in A1 PNs. (**D**) Left: Average effect of control (black) and ZX1 (red), an extracellular zinc chelator, on PN responses to broadband noise of different intensities [30- to 80-dB sound pressure level (SPL) sounds] in ZnT3-CreERT2^−/−^ /Rox^−/−^-mCherry mice [two-way analysis of variance (ANOVA), ZX1 effect, *P < 0.0001]. Right: Scatter plot of sound-evoked responses in control versus ZX1 in ZnT3-CreERT2^−/−^/Rox^−/−^-mCherry mice. Dashed line represents unity. (***P = 0.0002, *n* = 5 mice, permutation test). (**E**) Left: Same as in (D, left) but in ZnT3-CreERT2^+/+^/Rox^+/+^-mCherry mice (two-way ANOVA, ZX1 effect, *P* = 0.9439). Right: Same as in (D, right) but in ZnT3-CreERT2^+/+^/Rox^+/+^-mCherry mice (*P* = 0.94, *n* = 6 mice, permutation test). Table S1 shows the statistical details.

To further confirm the ability of DreO-mediated recombination to induce functional deletion of *Znt3* and expression of the mCherry reporter in these mice, we injected ZnT3- CreERT2^+/+^/Rox^+/+^-mCherry and ZnT3-CreERT2^−/−^/ Rox^−/−^-mCherry mice with AAV.Syn.DreO to express DreO specifically in neurons and AAV.CaMKII.GCaMP6f to express the green fluorescent protein-based genetically encoded calcium indicator (GCaMP6f) specifically in PNs into the AC (Fig. 2A). To assess whether DreO-mediated recombination induced mCherry expression, we used an anti-mCherry antibody in AC-containing fixed brain sections from mice injected with both viruses (Fig. 2B). We detected GCaMP6f expression in both ZnT3-CreERT2^+/+^/Rox^+/+^-mCherry and ZnT3- CreERT2^−/−^/Rox^−/−^-mCherry mice, but only ZnT3-CreERT2^+/+^/ Rox^+/+^-mCherry mice showed selective expression of mCherry fluorescence in the AC (Fig. 2B). To confirm the functional loss of ZnT3 in mCherry-positive cells, we performed in vivo transcranial wide-field GCaMP6f calcium imaging in awake mice 3 to 4 weeks after AAV injections (Fig. 2C). We relied on an approach we have used in prior studies, where application of ZX1, an extracellular, fast, high-affinity zinc-specific chelator (*8*, *30*), decreased responses to sound in primary AC (A1) PNs in wild-type (WT) mice but had no effect in constitutive ZnT3 KO mice (*13*, *14*). When we plotted the sound-evoked responses before and after ZX1 infusion, we found, as expected, that ZX1 decreased the PN responses to sound in ZnT3-CreERT2^−/−^/Rox^−/−^-mCherry mice (Fig. 2D), indicating that synaptic zinc signaling enhances the PN responses in A1. In contrast, we found that the effect of ZX1 was eliminated in ZnT3-CreERT2^+/+^/Rox^+/+^-mCherry mice (Fig. 2E), consistent with constitutive ZnT3 KO mice. These results demonstrate the functional loss of ZnT3 in AC neurons and validate the DreO-mediated recombination.

### A previously unknown thalamocortical zincergic pathway modulates NMDARs in AC L5 PNs

**Fig. 3.**
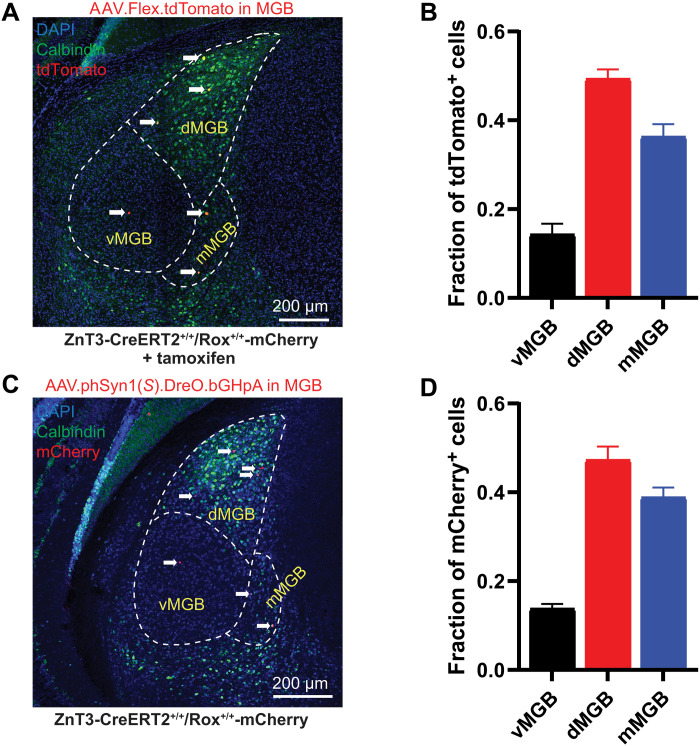
ZnT3-CreERT2/Rox mice reveal that a subset of thalamic neurons express ZnT3. (**A**) Representative stitched MGB image of coronal section from ZnT3-CreERT2^+/+^/Rox^+/+^-mCherry mice injected with AAVs in MGB to label ZnT3-expressing cells (AAV.Flex.tdTomato) and treated with tamoxifen. Anti-calbindin labeling (green) was used to determine MGB subregions. White arrows indicate tdTomato-labeled cells. (**B**) Average fraction of tdTomato-labeled cells by MGB subregion corresponding to (A) (means ± SEM; *n* = 4 mice, 10 slices per mouse). (**C**) Representative stitched MGB image of coronal section from CreERT2^+/+^/Rox^+/+^-mCherry mice injected with AAV in MGB to delete ZnT3 from MGB neurons [AAV.phSyn1(*S*).DreO.bGHpA] showing mCherry expression after Dre recombination. Anti-calbindin labeling (green) was used to determine MGB subregions. White arrows indicate mCherry-labeled cells. (**D**) Average fraction of mCherry-labeled nuclei by MGB subregion corresponding to (C) (means ± SEM; *n* = 3 mice, 10 slices per mouse).

Histochemical approaches to date have revealed a zincergic band in L1 of the neocortex (*27*, *31*). However, the cellular origin of the terminals that form this band is unknown. Given that L1 receives strong thalamic input (*32*, *33*), we hypothesized that the thalamus may be one source of these L1 zincergic terminals in the AC. While previous studies have shown that thalamic neurons are lacking of or express ZnT3 only transiently during early development (*31*, *34*–*36*), the techniques and tools used to date are neither able to label all zincergic cell bodies nor can they assess whether identified zincergic neurons form functional modulatory synapses (*26*). To address these limitations and determine whether the thalamus sends functional zincergic synapses to the AC, we injected ZnT3-CreERT2^+/+^/Rox^+/+^-mCherry mice with AAV.Flex.tdTomato into the auditory thalamus [medial geniculate body (MGB)] to express tdTomato in ZnT3-positive neurons, and we treated the mice with tamoxifen for 5 days. Thirty days after injection, we examined these mice for tdTomato expression (red) in the MGB. We found tdTomato-positive zincergic cells in the MGB (Fig. 3A). To evaluate the localization of zincergic cells in the MGB, we used an anti-calbindin antibody, which preferentially labels the medial and dorsal but not the ventral MGB (*37*). We found that zincergic cells were mostly distributed in the dorsal (dMGB) and medial (mMGB) divisions of the nucleus (Fig. 3, A and B). We did not observe any labeled zincergic cells in ZnT3-CreERT2^+/+^/Rox^+/+^-mCherry mice that were injected with the same AAV in the absence of tamoxifen or in ZnT3-CreERT2^−/−^/Rox^−/−^-mCherry mice treated with tamoxifen (fig. S3, A and B). We additionally evaluated the expression pattern of mCherry labeling following DreO/Rox-mediated recombination in the MGB. ZnT3-CreERT2^+/+^/Rox^+/+^-mCherry mice injected with AAV.Syn.DreO into the MGB revealed mCherry labeling in a similar pattern to that of the tdTomato expression, with a higher proportion of labeled cells in the medial and dorsal regions as compared to the ventral MGB (Fig. 3, C and D). We did not observe any mCherry-positive zincergic cells in ZnT3-CreERT2^−/−^/Rox^−/−^-mCherry mice injected with AAV.Syn.DreO (fig. S3C). Together, these results indicate that a subset of thalamic neurons are zincergic and mainly localized in the medial and dorsal regions of the MGB.

**Fig. 4.**
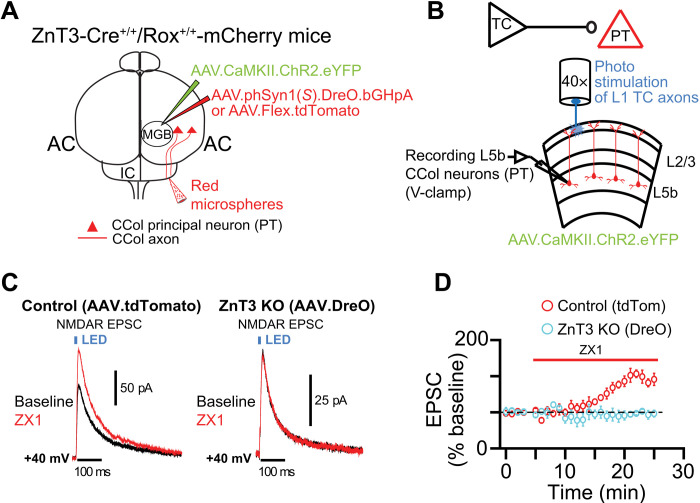
Functional zincergic thalamocortical synapses mediate zinc-mediated inhibition of NMDAR EPSCs in AC PT neurons in ZnT3-CreERT2/Rox mice. (**A**) Schematic illustration of stereotaxic injections in ZnT3-CreERT2^+/+^/Rox^+/+^ mice. AAV.CamKII.ChR2.eYFP (to express ChR2) and either AAV.phSyn1(*S*).DreO.bGHpA (for ZnT3 KO) or AAV.Flex.tdTomato (control for the viral injection) were injected into the MGB. Red microspheres were injected into the IC to label PT neurons in AC. (**B**) Schematic illustration of the brain slice electrophysiology experiment. L1 terminals of thalamocortical (TC) axons were stimulated in AC with blue light while recording from PT neurons in L5b. (**C**) Left: Representative traces of AC PT NMDAR Lev-EPSCs before (baseline, black) and after ZX1 (red) in ZnT3 control (AAV.Flex.tdTomato–injected) mice. Right: Same as in left panel but in ZnT3 KO (AAV DreO–injected) mice. (**D**) Time course of the average amplitude of NMDAR Lev-EPSCs before and after ZX1 in ZnT3 control (red) and DreO ZnT3 KO (light blue) mice (*P* < 0.000, *n* = 6 cells from three control mice and *n* = 6 cells from three DreO ZnT3 KO mice; table S1). Error bars indicate ±SEM.

Next, we examined whether these MGB zincergic neurons form functional synapses in the AC. To achieve this, we injected the MGB of ZnT3-CreERT2^+/+^/Rox^+/+^-mCherry mice with AAV.CamKII.ChR2.eYFP to express Channelrhodopsin (ChR2) in MGB PNs, along with AAV.Syn.DreO to express DreO specifically in neurons to delete ZnT3 (Fig. 4A) or AAV.Flex.tdTomato to control for potential effects of the viral injection. Thalamic axons that terminate in L1 mostly make contacts with the ascending apical dendritic tufts of L5 PNs. Το test whether thalamocortical synapses to L5 PNs neurons are modulated by synaptic zinc released by thalamic neurons, we performed in vitro brain slice electrophysiology experiments. To test this hypothesis, we targeted the L5 PT neurons, in which CCols are a subtype (*38*); it is known that PTs extend their dendritic arborizations into L1 of the cortex, including AC, where they receive thalamic input (*32*, *39*). After labeling PT CCols by injecting red retrograde beads into the IC and localizing the AC (*29*), we applied a single pulse of blue focal light onto L1 to elicit NMDAR light evoked (Lev)–excitatory postsynaptic current (EPSCs) from labeled L5 PTs (Fig. 4, B and C). When we injected the control virus (AAV.Flex.tdTomato) into the MGB, we found that ZX1 enhanced the amplitude of NMDAR Lev-EPSCs, supporting the notion that thalamic zincergic synapses are functional and that synaptic zinc inhibits NMDAR EPSCs in PTs [Fig. 4, C (left) and D]. The ZX1 effect was eliminated when AAV.Syn.DreO was injected to delete ZnT3 in MGB zincergic neurons [Fig. 4, C (right) and D], suggesting that synaptically released zinc from ascending thalamic neurons inhibits NMDAR EPSCs in AC PT neurons. This is an important finding demonstrating previously unknown thalamic modulation of AC PT neurons by synaptic zinc released from MGB neurons.

### Cell type–specific *Znt3* expression and cell type– and synapse-specific zincergic signaling in AC

**Fig. 5.**
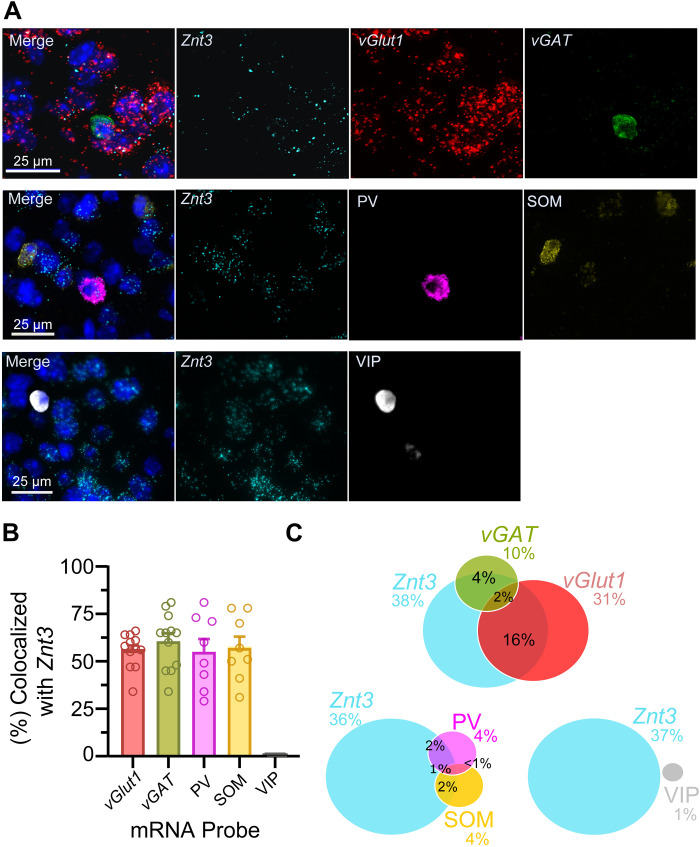
FISH studies reveal cell type–specific *Znt3* expression in AC. (**A**) Representative images from FISH experiments in AC L2/3 demonstrating colocalization of mRNA probes with DAPI stain (blue). *Znt3*, cyan; *Vglut1*, red; *vGAT*, green; PV, pink; SOM, yellow; VIP, white. (**B**) Bar graph showing the proportion *of vGlut1*, *vGAT*, PV, SOM, or VIP cells that express *Znt3*. Data points represent one section as means ± SEM (*n* = 3 to 4 mice, two sections per mouse). (**C**) Venn diagrams illustrating the average proportions of all AC cells expressing distinct mRNA markers (*ZnT3*, *vGlut1*, *vGAT*, PV, SOM, and VIP). Data are mean (*n* = 3 to 4 mice, two sections per mouse). The total number of cells counted in each neuronal population ranged from 1612 to 3347 per hemisphere. The following combination of probes were used with *Znt3*: *Vglut* and *Vgat* (26,481 cells counted), PV and SOM (19,271 cells counted), and VIP (29,563 cells counted). Total cells (75,315) were sampled across all probe sets.

ZnT3 is strongly expressed in the AC (*27*); however, the molecular makeup and proportion of ZnT3-expressing glutamatergic or GABAergic neurons in this region remains unknown. To address this, we used RNAscope FISH to quantify cell subtype density (Fig. 5A) in AC L2/3 of adult WT mice. We found that *Znt3* mRNA was expressed in 55.7% of glutamatergic cells and 60.5% of GABAergic cells (Fig. 5B), as demonstrated by colocalization of punctate nuclear labeling with vesicular glutamate transporter 1 (*Vglut1*) or vesicular GABA transporter (*Vgat*) mRNA. Next, we sought to determine which GABAergic cell population expressed *Znt3*, and we found that *Znt3* mRNA was present in half of the PVs and SOMs (55 and 57%, respectively) but absent in vasoactive intestinal polypeptide-expressing (VIP) cells (Fig. 5B). We also quantified the average proportions of all AC L2/3 cells and found that *Znt3*-positive cells represent 38% of the neuronal population in the AC. Approximately half of *Znt3*-positive cells consisted of glutamatergic cells (16% of all cells) and GABAergic cells (4% of all cells; Fig. 5C). It is possible that the remaining half of the zincergic cells did not colocalize with either *Vglut1* or *Vgat*, because the mRNA levels detected were too low for quantification by the methods used in the study. Another possibility is that many zincergic cells may be non-neuronal. Notably, when evaluating *Znt3* expression with interneuronal subtypes, *Znt3* was coexpressed with PV in 2% of cells and with SOM in 2% of cells but was not coexpressed in VIP cells (Fig. 5C). In summary, these results define the cell type–specific pattern of *Znt3* expression in AC L2/3.

**Fig. 6.**
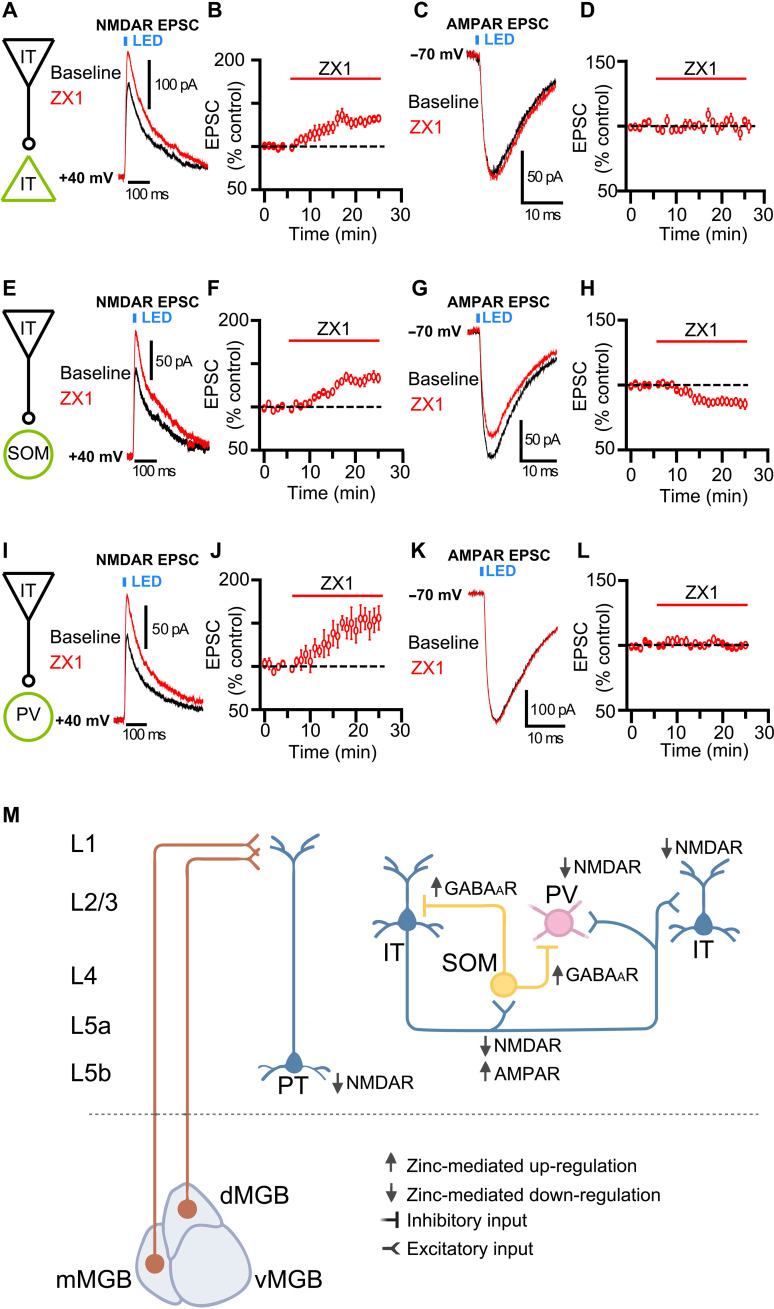
Identified zincergic synapses and their targets within AC L1 to L3. (**A** to **D**) Zincergic synapses between IT pyramidal neurons in AC L2/3. (A) Left: Synapse illustration. Right: Representative traces of L2/3 IT NMDAR Lev-EPSCs in baseline (black) and after ZX1 (red). (B) Time course of the average amplitude of NMDAR Lev-EPSCs before and after ZX1. Average effect of ZX1 normalized to baseline (*P* = 0.007, *n* = 7 cells from five mice). (C) Representative traces of AMPAR Lev-EPSCs in baseline (black) and after ZX1 (red). (D) Same as in (B) but for AMPAR (baseline versus ZX1: *P* = 0.237, *n* = 7 cells from four mice). (**E** to **H**) Zincergic synapses between IT pyramidal neurons and SOM interneurons in AC L2/3. (E) Left: Synapse illustration. Right: Representative traces of NMDAR Lev-EPSCs in baseline (black) and after ZX1 (red). (F) Same as in (B) but for SOM (*P* = 0.003, *n* = 6 cells from six mice). (G) Representative traces of AMPAR Lev-EPSCs in baseline (black) and after ZX1 (red). (H) Same as in (F) but for AMPAR (*P* = 0.011, *n* = 10 cells from 10 mice). (**I** to **L**) Zincergic synapses between IT pyramidal neurons and PV interneurons in AC L2/3. (I) Left: Synapse illustration. Right: Representative traces of NMDAR Lev-EPSCs in baseline (black) and after ZX1 (red). (J) Same as in (B) but for PV (*P* = 0.015, *n* = 5 cells from five mice). (K) Representative traces of AMPAR Lev-EPSCs in baseline (black) and after ZX1 (red). (L) Same as in (J) but for AMPAR (*P* = 0.388, *n* = 4 cells from four mice). (**M**) Cartoon illustrating zincergic synapses and their targets in AC L1 to L3. For statistical analysis, we compared the baseline amplitude 5 min before ZX1 against the amplitude of the last 5 min (means ± SEM; table S1).

Next, we investigated how cell type–specific expression of *Znt3* affects synaptic zinc signaling in L2/3 synapses of AC. *Znt3* mRNA is expressed in both PV and SOM neurons, yet synaptic zinc enhances GABA inhibitory postsynaptic currents (IPSCs) mediated by SOM, but not PV neurons in the AC (*12*), suggesting that *Znt3* mRNA expression does not always result in functional zincergic neuromodulation, at least of GABAergic IPSCs. Thus, although our gene expression data revealed *Znt3* expression in AC L2/3 glutamatergic neurons, we next tested whether synaptic zinc release modulates neurotransmission in AC L2/3 glutamatergic synapses. To achieve this, we selectively expressed ChR2 in glutamatergic PNs by injecting calcium/calmodulin-dependent protein kinase II (CaMKII)–dependent ChR2 AAV viral vectors into the AC of WT, SOM–green fluorescent protein (GFP), or PV-GFP mice (see Materials and Methods). To label glutamatergic corticocortical PNs (ITs; see the “Labeling of IT and PT PNs” section), we injected green fluorescent retrograde microspheres into the contralateral AC. After localizing AC, we elicited NMDAR or AMPAR Lev-EPSCs by stimulating intracortical excitatory afferents with a single pulse of blue light applied to AC L2/3 and recorded from adjacent AC L2/3 ITs (PNs) in WT mice, GFP-labeled SOMs in SOM-GFP mice, and GFP-labeled PVs in PV-GFP mice. We used ZX1 to assess zincergic modulation of glutamatergic neurotransmission in L2/3 AC. We found that ZX1 enhanced NMDAR Lev-EPSCs in L2/3 ITs (Fig. 6, A and B), SOMs (Fig. 6, E and F), and PVs (Fig. 6, I and J), suggesting that synaptic zinc inhibits NMDAR EPSCs in L2/3 PNs, SOMs, and PVs. In conventional ZnT3 KO mice (see the “Animals” section), we found that ZX1 did not enhance NMDAR Lev-EPSCs in either L2/3 ITs (fig. S4, A and B) or SOMs (fig. S4, C and D), suggesting that it is the vesicular, synaptic zinc that mediates this zinc-mediated modulation. Moreover, ZX1 inhibited AMPAR Lev-EPSCs only in SOMs (Fig. 6, G and H) without affecting AMPAR Lev-EPSCs in either ITs (Fig. 6, C and D) or PVs (Fig. 6, K and L), suggesting that synaptic zinc enhances AMPAR EPSCs only in SOMs. Figure 6M illustrates the identified zincergic synapses and their targets within AC L1 to L3.

## DISCUSSION

Here, we developed and validated the ZnT3-CreERT2/Rox-mCherry mouse using RNAscope FISH and found selective *Cre* mRNA expression in zincergic neurons. We confirmed that the expressed CRE recombinase in zincergic neurons was functional because it enabled Cre-dependent expression of tdTomato in the AC and MGB. We also validated cell type–specific DreO-mediated recombination in ZnT3 Cre^+/−^/Rox^+/−^::PV-Dre^+/−^ mice. Moreover, we took advantage of the DreO-Rox system to delete the *Znt3* gene in MGB neurons using DreO recombinase–containing AAVs. We used this novel mouse tool to demonstrate that, contrary to prior findings, there are zincergic neurons in the thalamus (MGB). These neurons project to L1 AC and release synaptic zinc that inhibits NMDAR EPSCs in AC PT neurons.

Although recent findings have advanced understanding of network organization and cell type–specific connectivity in thalamocortical connections (*33*), the neuromodulatory- and activity-dependent changes on the mode of operation of these connections remain less understood. Thus, the discovery of this thalamocortical zincergic modulation will advance the field to a new level of understanding about thalamocortical neuromodulatory mechanisms and create a new framework for understanding thalamocortical models and data. The role of thalamocortical zincergic modulation in sound processing is unknown; however, it is interesting that these zincergic thalamocortical synapses originate mainly from the nonprimary auditory (nonlemniscal) divisions of the auditory thalamus (dMGB and mMGB; Fig. 3). Although the exact role of these nonlemniscal nuclei is unclear, it is known that they send and receive projections among a wide range of brain centers such as midbrain-, cortex-, and limbic-related sites, likely affecting higher cognitive functions, such as internal state and multisensory integration and perception (*40*). However, two limitations should be considered in interpreting these results. Namely, variation in viral spread could contribute to a bias in the observed distribution of labeled cells. However, we think that this is unlikely to explain the results, as we used substantial volumes and high viral titers of AAV serotype 9, which is known to spread widely across an injection area and infect a diverse range of cells in the brain (see the “General comment on viral injections” section). In addition, it is possible that the use of different viruses or the use of two recombination systems (CreERT2 versus DreO) may affect the expression pattern of the reporter gene and thus lead to layer or subregion expression differences. However, the consistent subregion labeling of ZnT3-positive neurons in the MGB when using either the CreERT2 or DreO recombinase system (Fig. 3, A and B versus C and D) suggests that the two recombination systems and associated viruses induce similar expression patterns of the reporter genes. In summary, given the emerging modulatory role of synaptic zinc signaling in synaptic transmission, short- and long-term synaptic plasticity, and A1 sound processing (*3*) and the importance of cortico-thalamo-cortical loops in consciousness and perception (*33*), it is essential for future studies to integrate the zinc-dependent contributions with the larger fields of neuromodulation and plasticity of auditory and multisensory perception.

Toward achieving this goal, the novel mouse line we developed will permit the use of combinatorial/intersectional approaches to further characterize the anatomical, physiological, and behavioral roles of ZnT3 and synaptic zinc signaling in auditory networks and in many other brain areas where zincergic signaling is present (*31*). Future studies using the simultaneous combination of Cre-Lox and DreO-Rox systems in the same mouse will be helpful for more detailed characterization of zincergic modulation and other targeted loci, thus expanding the toolbox of techniques that permit these precise and detailed analyses.

Moreover, to address the aforementioned limitations of the global ZnT3 KO mice and the potential specificity issues of ZX1 infusion in the cortex, especially given our validation of the cell type–specific DreO-mediated recombination in ZnT3 Cre^+/−^/ Rox^+/−^::PV-Dre^+/−^ mice (fig. S2), in future studies, we plan to use these newly developed lines and DreO viruses with cell type–specific promoters in combination with ZnT3-CreERT2/Rox-mCherry mice to investigate the cell type–specific roles of the broadly distributed zincergic neurons in the AC. These combinatorial approaches will enable us to determine the causal and cell type–specific complex role of zincergic neurons in synaptic transmission, sound processing, cortical adaptation to changes in background sound stimulus statistics, and cortical plasticity.

One potential limitation with our transgenic mouse is that it might lead to abnormal or ectopic expression and function of ZnT3. However, consistent with previous in vitro studies using WT mice (*8*, *11*, *18*), our electrophysiological data using the ZnT3-CreERT2^+/+^/Rox^+/+^-mCherry mice demonstrate that the zinc chelator ZX1 enhanced the amplitude of NMDAR Lev-EPSCs (Fig. 4, C and D). Furthermore, consistent with previous in vivo studies using WT mice (*13*), our in vivo calcium imaging data demonstrated that the application of ZX1 decreased responses to sound in A1 PNs (Fig. 2D). These data are consistent with normally functioning zinc signaling in our transgenic mice.

An additional potential limitation with our transgenic mouse is that previous studies have suggested occasional crossover recombination between Cre and Rox sites (*28*, *41*). If there had been crossover recombination, then CreERT2 would recombine the Rox sites, and we would see *mCherry* expression in the absence of *DreO*. However, we found no *mCherry* expression in the absence of *DreO* expression (Fig. 1I), suggesting the lack of crossover recombination between Cre and Rox sites. Together, the ZnT3-CreERT2/Rox-mCherry mouse provides a unique opportunity to map the distribution of all zincergic neurons and circuits in the mouse brain using genetic and viral tools and allows for conditional deletion of *Znt3* in a cell/region-specific manner.

Consistent with previous studies (*5*), our RNAscope FISH experiments confirmed that, in the AC, most of zincergic cells are glutamatergic. We found that nearly half of *Vgat* positive neurons were zincergic and, therefore, are GABAergic interneurons, suggesting that this subset of zincergic neurons corelease zinc and GABA, which is consistent with previous electrophysiological studies, where synaptic zinc enhances SOM-mediated GABAergic inhibition in AC (*12*). In the neocortex, GABAergic neurons are chemically and functionally heterogeneous. Thus, we sought to elucidate the molecular phenotype of zincergic interneurons in the AC. We found that a subset of zincergic interneurons express SOM or PV mRNA. Our results are consistent with previous transcriptomic studies, which show that cortical IT, PV, and SOM, but not VIP, neurons express the *Znt3* gene (*42*–*45*) (also 2020 Allen Institute for Brain Science, Allen Cell Types Database: RNA-Seq Data; https://portal.brain-map.org/atlases-and-data/rnaseq).

It is also interesting that the ZnT3 gene has emerged as a major marker for different PN subtypes (*43*), suggesting that *Znt3* gene expression may segregate PNs by type and layer (*27*, *43*). Moreover, our results on the cell type– and synapse-specific zincergic signaling in the AC and our previous work (*12*–*14*, *20*) are consistent with recent results from cortical synapses in the somatosensory cortex, where zinc modulation of NMDARs is also cell type– and synapse-specific (*18*). Last, zinc neuromodulation is stimulus- and context-dependent and can act through complex circuit effects involving both excitatory and inhibitory neurons (*12*–*14*, *20*). For example, while the direct effect of synaptic zinc on PNs is to inhibit NMDARs (Figs. 4, C and D, and 6, A, E, and I), overall, synaptic zinc increases the sound-evoked responses in AC PNs (Fig. 2D), likely by decreasing the responses of PV and SOM interneurons (*13*). Our results support a crucial and previously unknown role of synaptic zinc signaling in cortical and thalamocortical circuits, thus expanding the neuromodulatory role of synaptic zinc signaling in the brain.

In summary, here, we present the development, validation, and application of a new genetic mouse strategy to an important biological question and demonstrate its superiority over existing approaches and techniques. By using this new tool, we unmasked previously unknown features of thalamocortical modulation by synaptic zinc.

## MATERIALS AND METHODS

### Animals

All mice handling was approved by the Institutional Animal Care and Use Committee at the University of Pittsburgh and National Institute on Drug Abuse (NIDA). The approved Institutional Animal Care and Use Committee (IACUC) protocol numbers that were used for this study were #17071036, #17127808, and 19-NRB-36. Male and female mice between the ages of postnatal day 28 (P28) and P65 were used for experiments (see experimental methods subsections for specific age details during each experimental manipulation/step). In general, mice were injected at the age of P28 to P35. Electrophysiological recordings from AC-injected mice were performed at least 12 days after injection at a minimum age of P40, and recordings from MGB-injected mice were performed 30 days after injection at a minimum age of P58. Developmental studies in rodents indicate that the maturation of synaptic, cellular, and network properties and glutamate receptor subunit composition in AC and MGB are completed by P29 (*46*–*48*), concurrently with changes in A1 frequency tuning (*49*, *50*). Moreover, cortical inhibition and excitation are cotuned by P21 (*48*, *50*). Thus, at the time of recording, all mice were older than P29, when the AC and MGB are fully mature and, thus, the age groups we used in our studies did not differ in their cellular, synaptic, and network baseline properties. Moreover, ZnT3 expression intensifies in the neocortex, hippocampus, amygdala, and parts of the entorhinal and perirhinal cortices throughout early postnatal development and maximizes by P28 (*7*). Thus, the age groups we are using have adult zincergic innervation. Male and female ZnT3-CreERT2/Rox-mCherry mice (inGenious Targeting Laboratory) were used for experiments shown in Figs. 1 to 4. The experimental mice, referred to as ZnT3-CreERT2^+/+^/Rox^+/+^-mCherry, and the control mice, referred to as ZnT3-CreERT2^−/−^/Rox^−/−^, that we used for these experiments are genotyped littermate mice born from heterozygous breeding pairs. ZnT3-CreERT2^−/−^/Rox^−/−^-mCherry mice are therefore WT littermates. Male and female ZnT3-CreERT2/Rox^+/−^::PV-Dre^+/−^ mice for experiments in fig. S2 were generated by breeding ZnT3-CreERT2^+/+^/Rox^+/+^-mCherry mice with PV-Dre^+/+^ mice (strain code #021190, the Jackson Laboratory). Male and female ZnT3-CreERT2/Rox-mCherry and C57BL/6 J mice (strain code #000664, the Jackson Laboratory) were used for experiments shown in Fig. 5. Male and female ICR (Envigo) mice were used for experiments in Fig. 6 (A to D). SOM-GFP (GIN) mice (*51*) (stock #003718, the Jackson Laboratory, Bar Harbor, ME), were used for experiments shown in Fig. 6 (E to H). Male and female PV-GFP mice (*52*) (stock #007677, the Jackson Laboratory, Bar Harbor, ME), were used for experiments shown in Fig. 6 (I to L). Male and female ZnT3 WT or KO mice (*4*) (stock #005064, the Jackson Laboratory, Bar Harbor, ME) were used for experiments shown in fig. S4 (A and B). ZnT3 KO mice lack ZnT3 and thus synaptic zinc (*4*). Male and female SOM-GFP Het/ZnT3 Het or SOM-GFP Het/ZnT3 KO mice, used in fig. S4 (C and D), were generated by crossing ZnT3 KO mice with the F1 generation of the ZnT3 KO x SOM-GFP cross (the Jackson Laboratory, Bar Harbor, ME). This crossing resulted in littermate SOM-GFP mice heterozygous for ZnT3 (SOM-GFP/ZnT3 Het), which express ZnT3 and synaptic zinc (*4*), and SOM-GFP/ZnT3 KO mice, which lack ZnT3 and synaptic zinc. In ZnT3 heterozygous mice, ZnT3 expression is present on all synaptic vesicles and the vesicular zinc levels were approximately 50% reduced compared to the WT (*4*). However, our results here and our previous work (*12*) support that the expressed ZnT3 and, thus, the available zinc in heterozygous mice were enough for the observed biological effects.

### Targeting vector

The ZnT3-CreERT2^+/+^/Rox^+/+^-mCherry targeting vector (Fig. 1A and fig. S1) was designed so that the 5′ short homology arm was 3.0 kb and the 3′ long homology arm was 6.6 kb. Recombinant clones were generated from electroporation of the targeting vector into IC1 (C57BL/6) embryonic stem cells (inGenious Targeting Laboratory, Ronkonkoma, NY) and confirmed by polymerase chain reaction and sequencing analyses. Blastocyst injection of recombinant clones was performed to produce chimeric mice, which were bred to flippase (FLP) deleter mice to remove the neomycin cassette. Germline transmission mice were confirmed for Neo deletion.

### Stereotaxic injections for in vitro electrophysiology

Male or female mice were anesthetized with inhaled isoflurane (induction, 3% in oxygen; maintenance, 1.5% in oxygen) and secured in a stereotaxic frame (Kopf, Tujunga, CA). Mice were between P28 and P35 at the time of surgery. Core body temperature was maintained at ~37°C with a heating pad and eyes were protected with ophthalmic ointment. Lidocaine (1%) was injected under the scalp, and an incision was made into the skin at the midline to expose the skull.

#### Labeling of IT and PT PNs

PNs (PTs or CCols and ITs or corticocallosal) in the AC, in ZnT3-CreERT2^+/+^/Rox^+/+^-mCherry, ICR and ZnT3 WT or KO and SOM-GFP Het/ZnT3 Het or SOM-GFP Het/ZnT3 KO mice, were retrogradely labeled by injecting green or red fluorescent latex microspheres (Lumafluor) in the contralateral AC to label ITs (in a small craniotomy drilled 4 mm posterior to bregma and 4 mm lateral, injection depth of 0.4 to 0.8 mm) and the ipsilateral IC to label PTs (1 mm posterior to lambda and 1 mm lateral, injection depth of 0.75 mm). Note that fluorescent retrograde bead injections into the IC also label neurons in deep L6 that correspond to approximately 10% of the projecting CCols in ipsilateral IC (*53*–*56*), but this L6-labeled cellular population does not contribute to the data generated in this study. A volume of ~0.12-μl microspheres was pressure-injected (25 psi, 10- to 15-ms duration) from capillary pipettes (Drummond Scientific) with a Picospritzer (Parker Hannifin). Although a small proportion of the retrogradely labeled neurons that project to contralateral AC are PVs (*57*), we recorded from morphologically defined ITs according to their larger cell bodies and distinct shape, compared to the small and round-shaped PVs (*12*).

After injection, the pipette was held in the brain for 2 min before slowly withdrawing. To specifically activate AC PNs, ICR, ZnT3 WT, or KO mice were intracranially injected in right AC with recombinant AAV encoding CaMKII-dependent ChR2–enhanced yellow fluorescent protein (EYFP) fusion protein [AAV9.CaMKIIa-hChR2(H134R)-EYFP; titer of 8.96 × 10^13^ genome copies/ml; Addgene] at the same time with the injection of microspheres. SOM-GFP, SOM-GFP Het/ZnT3 Het or SOM-GFP Het/ZnT3 KO, and PV-GFP were injected with recombinant AAV encoding CaMKII-dependent ChR2-mCherry fusion protein [AAV9.CaMKIIa-hChR2(H134R)-mCherry; titer of 1 × 10^13^ genome copies/ml; Addgene]. A small craniotomy (~0.4 mm in diameter) was made over the temporal cortex (~4 mm lateral to lambda). A glass micropipette was backfilled with mineral oil and connected to a 5-μl glass syringe (Hamilton, 525 Reno, NV). After loading the viral vector, the micropipette was inserted into the cortex of 1 mm past the surface of the dura with a micromanipulator (Kopf). We used a syringe pump (World Precision Instruments, Sarasota, FL) to inject 400 nl of the virus solution [diluted 1:1 in phosphate-buffered saline (PBS)] over the course of 5 min, and the pipette was left in place for 2 min. The pipette was then removed, and the scalp of the mouse was closed with cyanoacrylate adhesive. Mice were injected with nonsteroidal anti-inflammatory drug carprofen (5 mg/kg; Henry Schein Animal Health) for 24 hours before and 48 hours after surgery. Mice were monitored for signs of postoperative stress and pain.

To stimulate thalamic afferents (Fig. 4, A to D), ZnT3-CreERT2^+/+^/Rox^+/+^-mCherry mice were injected in right MGB with viral solution of AAV encoding CaMKII-dependent ChR2-EYFP fusion protein [AAV9.CaMKIIa-hChR2(H134R)-EYFP; titer of 8.96 × 10^13^ genome copies/ml; Addgene]. A small craniotomy (~0.4 mm in diameter) was made 3 mm posterior to bregma and 2 mm lateral to midline. A glass micropipette, containing the viral vector, was inserted 3 mm past the surface of the skull with a micromanipulator (Kopf). We injected 500 nl of the virus solution (undiluted) over the course of 5 min. The pipette was left in place for 20 min after the end of each injection.

Although there is evidence supporting CaMKII expression also in inhibitory neurons, it is also evidenced that glutamate decarboxylase (GAD)-positive neurons represent only ∼1% of medial geniculate neurons (*58*, *59*), and they mostly exert a local inhibition without giving rise to projection toward AC; thus, the CaMKII-expressing thalamocortical neurons are likely projection excitatory neurons. However, to isolate the NMDAR-mediated excitatory neurotransmission, AMPAR and GABA type A receptor (GABA_A_R) blockers were applied (Figs. 4, A to D, and 6, A, B, E, F, I, and J; and fig. S4). In addition, we applied tetrodotoxin to further ensure the study of monosynaptic excitatory inputs.

For ZnT3 ablation (ZnT3 KO condition), ZnT3- CreERT2^+/+^/Rox^+/+^-mCherry mice were injected in right MGB with recombinant AAV encoding phSyn1(*S*)-dependent DreO fusion protein [AAV5.phSyn1(*S*)-DreO-bGHpA; titer of 1 × 10^13^ genome copies/ml; Addgene]. Ablation of ZnT3 leads to mCherry expression (red). For control condition (no deletion of ZnT3), ZnT3-CreERT2^+/+^/Rox^+/+^-mCherry mice were injected in the right MGB with recombinant AAV encoding Cre-dependent tdTomato fusion protein (AAV9.Flex.tdTomato; titer of 1 × 10^13^ genome copies/ml; Addgene). These mice were treated with tamoxifen (75 mg/kg) for five consecutive days before euthanizing and slicing to allow the Cre-driven expression of tdTomato protein. We used a syringe pump (World Precision Instruments, Sarasota, FL) to inject separately 500 nl of each virus solution [undiluted for either AAV9.CaMKIIa-hChR2(H134R)-EYFP or AAV5.phSyn1(*S*)-DreO-bGHpA] over the course of 5 min. The pipette was left in place for 20 min after the end of each injection. Two hundred nanoliters (diluted 1:1 in PBS) of AAV9.Flex.tdTomato was injected under the control condition.

### Stereotaxic injections for anatomy

Male or female (P28 to P35 at the time of surgery) ZnT3-CreERT2^+/+^/Rox^+/+^-mCherry and ZnT3-CreERT2^−/−^/ Rox^−/−^-mCherry mice were treated as described above regarding the injection procedure. To label ZnT3-expressing neurons described in Fig. 1 (C to F), ZnT3-CreERT2^+/+^/Rox^+/+^-mCherry and ZnT3-CreERT2^−/−^/Rox^−/−^-mCherry mice were injected in the right AC with 400 nl (diluted 1:1 in PBS) of AAV9.Flex.tdTomato to label ZnT3-expressing neurons. Projection PNs (L5 PTs) in the AC were retrogradely labeled by injecting (0.12 μl) green-colored fluorescent latex microspheres (Lumafluor) in the ipsilateral IC. To label ZnT3-expressing neurons described in Fig. 3A and fig. S3, ZnT3-CreERT2^+/+^/ Rox^+/+^-mCherry and ZnT3-CreERT2^−/−^/Rox^−/−^-mCherry mice were injected in the right MGB with 400 nl (diluted 1:1 in PBS) of AAV9.Flex.tdTomato, and the injection pipette was left in place for 20 min after the end of injection. All mice injected with AAV9.Flex.tdTomato were administered with tamoxifen (75 mg/kg) for five consecutive days and kept for one additional week before euthanizing to permit the Cre-driven expression of tdTomato. This induction paradigm was chosen on the basis of previous publications and recommendation by the Jackson Laboratory (www.jax.org/research-and-faculty/resources/cre-repository/tamoxifen) (*60*).

For ZnT3 ablation experiments described in Fig. 3C and fig. S3C, ZnT3-CreERT2^+/+^/Rox^+/+^-mCherry and ZnT3-Cre^−/−^/ Rox^−/−^-mCherry mice were injected in right MGB with 500 nl of (undiluted) recombinant AAV encoding phSyn1(*S*)-dependent DreO fusion protein [AAV5.phSyn1(*S*)-DreO-bGHpA; titer of 1 × 10^13^ genome copies/ml; Addgene], as described above.

To evaluate AAV.DreO-induced *mCherry* mRNA expression in *Znt3*-expressing cells, described in Fig. 1 (G to I), ZnT3-CreERT2^+/+^/Rox^+/+^-mCherry and ZnT3-CreERT2^−/−^/ Rox^−/−^-mCherry mice were injected in right AC with 500 nl of (undiluted) recombinant AAV encoding phSyn1(*S*)-dependent DreO fusion protein [AAV5.phSyn1(*S*)-DreO-bGHpA; titer of 1 × 10^13^ genome copies/ml; Addgene], as described above.

### Stereotaxic injections for in vivo imaging

ZnT3-CreERT2^+/+^/Rox^+/+^-mCherry and ZnT3-CreERT2^−/−^/ Rox^−/−^-mCherry mice between P28 and P36 were anesthetized with inhaled isoflurane (induction, 3% in oxygen; maintenance, 1.5% in oxygen) and secured in a stereotaxic frame (Kopf, Tujunga, CA). Core body temperature was maintained at ~37°C with a heating pad, and eyes were protected with ophthalmic ointment. Lidocaine (1%) was injected under the scalp, and an incision was made into the skin at the midline to expose the skull. Using a 27-gauge needle as a scalpel, a small craniotomy (~0.4 mm in diameter) was made over the temporal cortex (~4 mm lateral to lambda). Two glass micropipettes were backfilled with mineral oil and connected to a 5-ml glass syringe (Hamilton, Reno, NV). After loading AAV5.phSyn1(*S*)-DreO-bGHpA and AAV9.CaMKII.GCaMP6f.WPRE.SV40 viral vectors in each micropipette, they were inserted into the cortex of 0.5 to 0.7 mm past the surface of the dura with a micromanipulator, one after the another (Kopf). We used a syringe pump (World Precision Instruments, Sarasota, FL) to inject 500 nl of each viral vector (undiluted) over the course of 5 min. The pipettes were left in place for 2 min after the end of the injection. After the second injection, pipette was removed, and the scalp of the mouse was closed with cyanoacrylate adhesive. Mice were injected with nonsteroidal anti-inflammatory drug carprofen (5 mg/kg; Henry Schein Animal Health) for 24 hours before and 48 hours after surgery. Mice were monitored for signs of postoperative stress and pain.

#### General comment on viral injections

Although AAV injections may cause toxic effects at times, we did not observe any evidence of cellular loss in the injected areas. Moreover, our electrophysiological data after the AAV injections demonstrate that neurons were healthy as evidenced by the value and stability of series and input resistance during recordings (see the “Slice electrophysiology” section for details). Finally, the zinc chelator ZX1 enhanced the amplitude of NMDAR Lev-EPSCs (Fig. 4, C and D), validating healthy cellular (zinc) signaling mechanisms.

### In vivo imaging preparation

Twenty-one to 28 days after viral vectors injections (P49 to P64), mice were prepared for in vivo calcium imaging. Mice were anesthetized with inhaled isoflurane (induction, 3% in oxygen; maintenance, 1.5% in oxygen) and positioned into a head holder. Core body temperature was maintained at ~37°C with a heating pad, and eyes were protected with ophthalmic ointment. Lidocaine (1%) was injected under the scalp and an incision (~1.5 cm in length) was made into the skin over the right temporal cortex. The head of the mouse was rotated ~45° in the coronal plane to align the pial surface of the right temporal cortex with the imaging plane of the upright microscope optics. The skull of the mouse was secured to the head holder using dental acrylic (Lang, Wheeling, IL) and cyanoacrylate adhesive. A tube (the barrel of a 25-ml syringe or an SM1 tube from Thorlabs, Newton, NJ) was placed around the animal’s body to reduce movement. A dental acrylic reservoir was created to hold warm artificial cerebrospinal fluid (ACSF) over the exposed skull. In preparing the ACSF, we removed contaminating zinc by incubating with Chelex 100 resin (Bio-Rad, Hercules, CA) for 1 hour. Subsequently, we removed the Chelex by vacuum filtration and added high purity calcium and magnesium salts (99.995% purity; Sigma-Aldrich, St. Louis, MO). The solution contained 130 mM NaCl, 3 mM KCl, 2.4 mM CaCl_2_, 1.3 mM MgCl_2_, 20 mM NaHCO_3_, 3 mM Hepes, and 10 mM d-glucose (pH 7.25 to pH 7.35; ~300 mOsm). For better optical access of the AC, we injected lidocaine-epinephrine (2% lidocaine and 1:100,000 weight/volume epinephrine) into the temporal muscle and retracted a small portion of the muscle from the skull. Mice were then positioned under the microscope objective in a sound- and light-attenuation chamber containing the microscope and a calibrated speaker (ES1, Tucker-Davis Davis Technologies, Alachua, FL). Acoustic stimuli were calibrated with 6.35-mm microphone (Bru¨el and Kjær, Nærum, Denmark) placed at the location of the animal’s ear within the chamber.

### Transcranial wide-field imaging of A1

After the in vivo imaging preparation, we performed transcranial imaging to locate A1 in each mouse as described previously (*14*). We removed the isoflurane from the oxygen flowing to the animal and began imaging sound-evoked responses at least 10 to 15 min later. Sounds were delivered from a free-field speaker 10 cm from the left ear of the mouse (ES1 speaker, ED1 driver, Tucker-Davis Technologies), controlled by a digital to analog converter with an output rate of 250 kHz (USB-6229, National Instruments, Austin, TX). We used ephus (*61*) to generate sound waveforms and synchronize the sound delivery and image acquisition hardware. We presented 50- or 60-dB sound pressure level (SPL) and 6-kHz tones to the mouse while illuminating the skull with a blue light-emitting diode (LED) (nominal wavelength of 490 nm; M490L2, Thorlabs). We imaged the change in green GCaMP6f emission with epifluorescence optics (eGFP filter set, U-N41017, Olympus, Center Valley, PA) and a 4× objective (Olympus) using a cooled charge-coupled device camera (Rolera, Q-Imaging, Surrey, BC, Canada). Images were acquired at a resolution of 174 × 130 pixels (using 4× spatial binning, each pixel covered an area of 171.1 μm^2^ of the image) at a frame rate of 20 Hz to locate A1 in each mouse. To localize A1, we used 50- or 60-dB SPL and 6-kHz tones and normalized the sound-evoked change in fluorescence after sound presentation (Δ*F*) to the baseline fluorescence (*F*), where *F* is the average fluorescence of 1 s preceding the sound onset (for each pixel in the movie). We applied a two-dimensional, low-pass Butterworth filter to each frame of the Δ*F*/*F* movie and then created an image consisting of a temporal average of 10 consecutive frames (0.5 s) beginning at the end of the sound stimulus. This image indicated two sound-responsive regions corresponding to the low-frequency tonotopic areas of A1 and the anterior auditory field (AAF) (*14*).

After A1 localization, we reanesthetized the mouse with isoflurane and using a micromanipulator (Siskiyou, Grants Pass, OR), we inserted a glass micropipette backfilled with mineral oil and connected to a 5-μl glass syringe into the cortex at the edge of this craniotomy. The pipette contained ACSF including 100 μM ZX1 (an extracellular, high-affinity, fast, zinc-specific chelator) (*8*, *14*). Once the pipette was inserted into the cortex, we removed the isoflurane. At least 60 to 80 min later to allow for recovery from isoflurane, we presented sound stimuli (6- to 64-kHz broadband noise, 100-ms duration, 5-ms ramps at 30- to 80-dB SPL in 5-dB SPL increment level), while measuring the changes in GCaMP6 fluorescence. After recording the responses to different sounds (8 to 10 presentations of each sound level in random order), we began to infuse the ZX1 solution into the cortex at a rate of 30 nl/min for 20 min. After 20 min, we reduced the pump speed to 9 nl/min and remeasured the sound-evoked responses.

To analyze sound-evoked responses, a region of interest (ROI; 150 to 200 mm × 150 to 200 mm) over A1 was then used to quantify the sound-evoked responses to sounds. We averaged the fluorescent intensity from all pixels in the ROI for each frame and normalized the Δ*F* to the *F* of the ROI to yield Δ*F*/*F* responses. Δ*F*/*F* responses from 8 to 10 presentations of the same sound level were averaged. Response amplitude was the peak (50-ms window) of the transcranial response that occurred within 1 s of the sound onset.

### Immunohistochemistry

For all immunohistochemistry experiments, 30 days after viral vector injections (P58 to P65), mice were transcardially perfused with ice-cold 1× PBS, followed by 4% paraformaldehyde in 1× PBS (pH 7.4). Brains were extracted and postfixed for 2 hours in 4% paraformaldehyde. Brains were then submerged sequentially in 15% sucrose in 1× PBS for 24 hours and 30% sucrose in 1× PBS until they no longer floated in solution (typically 24 hours). Brains were then frozen on dry ice, embedded in Tissue-Tek optimum cutting temperature compound (OCT), and sliced on a cryostat to a thickness of 50 μm into 1× PBS with 0.01% sodium azide.

#### Cell type–specific expression of mCherry in PV neurons

Slices were permeabilized in 1× PBS containing 0.3% Triton X-100 (PBST) three times for 10 min each and then blocked in PBST + 5% normal goat serum (blocking buffer) at room temperature. Slices were then incubated in blocking buffer containing primary antibodies against mCherry (1:500; rat anti-mCherry, M11217, Thermo Fisher Scientific) and PV (1:1000; mouse IgG1 anti-PV, Swant 235) overnight at 4°C. On the second day, slices were washed in PBST three times for 10 min each and then incubated in blocking buffer containing secondary antibodies [1:500; goat anti-rat immunoglobulin G (IgG)–Alexa Fluor 594, A11007, Thermo Fisher Scientific; 1:1000; goat anti-mouse IgG1–Alexa Fluor 488, A21121, Thermo Fisher Scientific) and 4and 21Scientificentifiblocki (DAPI) (1:5000) for 2 hours at room temperature. Last, slices were washed in 1× PBS three times for 10 min each, mounted onto slides, and coverslipped (ProLong Antifade Diamond Mounting Medium).

#### Cre-dependent tdTomato labeling experiments

Slices were permeabilized in PBST three times for 10 min each and then blocked in PBST + 5% normal goat serum (blocking buffer) at room temperature. Slices were then incubated in blocking buffer containing primary antibodies calbindin (1:1000; mouse IgG1 anti-calbindin D-28K, Swant 300) overnight at 4°C. On the second day, slices were washed in PBST three times for 10 min each and then incubated in blocking buffer containing secondary antibodies (1:1000; goat anti-mouse IgG1–Alexa Fluor 488, A21121, Thermo Fisher Scientific) and DAPI (1:5000) for 2 hours at room temperature. Last, slices were washed in 1× PBS three times for 10 min each, mounted onto slides, and coverslipped (ProLong Antifade Diamond Mounting Medium).

#### DreO-Rox–dependent nuclear mCherry expression experiments

Slices were permeabilized in PBST three times for 10 min each and then blocked in PBST + 5% normal goat serum (blocking buffer) at room temperature. Slices were then incubated in blocking buffer containing primary antibodies against mCherry (1:500; rat anti-mCherry, M11217, Thermo Fisher Scientific) and calbindin (1:1000; mouse IgG1 anti-calbindin D-28K, Swant 300) overnight at 4°C. On the second day, slices were washed in PBST three times for 10 min each and then incubated in blocking buffer containing secondary antibodies (1:500; goat anti-rat IgG–Alexa Fluor 594, A11007, Thermo Fisher Scientific; 1:1000; goat anti-mouse IgG1–Alexa Fluor 488, A21121, Thermo Fisher Scientific) and DAPI (1:5000) for 2 hours at room temperature. Last, slices were washed in 1× PBS three times for 10 min each, mounted onto slides, and coverslipped (ProLong Antifade Diamond Mounting Medium). When not being imaged, brains, slices, and mounted slides were protected from light to preserve fluorescence. Unless otherwise stated, all washes and incubations occurred on a rocker.

### RNAscope FISH

For tissue collection, ZnT3-CreERT2^+/+^/Rox^+/+^-mCherry and ZnT3-CreERT2^−/−^/Rox^−/−^-mCherry mice (Fig. 1, B and G to I) or WT C57BL/6 mice (Fig. 5) were briefly anesthetized with isoflurane and decapitated, their brains being explanted and allowed to snap-freeze on a stage of foil cooled by dry ice. Brains were sectioned into 14-μm coronal slices via cryostat (Leica CM3050 S) and mounted onto cold SuperFrost Plus charged slides (VWR). Two to three sections per subject and region of interest (*n* = 4) were taken 56 μm apart beginning approximately at bregma of −2.54 mm. Sections were briefly thawed to allow tissue adherence and allowed to rest at −20°C for 1 hour. Slides were stored at −80°C until stained. For staining, RNAscope in situ hybridization multiplex fluorescent V2 assay was performed on sections according to the protocol provided by ACDBio for fresh-frozen sections. A table of all probes used is shown in Table 1. Channels were counterstained with 520-, 570-, or 690-nm Opal Dyes (Akoya Biosciences). Sections were DAPI-counterstained and coverslipped using Fluoromount-G medium (Southern Biotech). Sections for quantification were imaged either on a Keyence B*Z*-X710 microscope at ×40 magnification or a Zeiss confocal (LSM700) microscope at ×20 magnification. Masks to isolate quantification to the AC were generated in ImageJ with the DAPI channel only, referencing the Paxinos Mouse Brain Atlas. Colocalization of mRNA probes was computed by the coincidence of mRNA puncta within individual DAPI ROIs, which were generated and computed using CellProfiler (*62*) (Fig. 5 with the percent counts) or in ImageJ (Fig. 1).

**Table 1.** mRNA probe names, targets, and catalog numbers for RNAscope experiments described in Figs. 1 and 5.

Gene code	mRNA	Catalog no.
CRE		312281
ZnT3	Mm-Slc30a3-C2	496291-C2
DRE		113861-C1
mCherry	mCherry	431201-C3
vGlut1	Mm-Slc17a7-C1	416631-C1
vGAT	Mm-Slc32a1-C3	319191-C3
PV	Mm-Pvalb	421931
SOM	Mm-Sst-C3	404631-C3
VIP	Mm-Vip	415961

### Confocal image acquisition and analysis for immunohistochemistry

Confocal images were captured using a Nikon A1R HD252 confocal microscope using Nikon’s NIS-Elements software. Areas of interest were identified visually initially using a 10× air objective. Once the target regions were determined, using a 20× air objective, Z-stacks were collected (0.5-μm steps, 1 zoom, pinhole dimensions: 1.2 airy units). Fields of view with 10% *x*-*y* overlap were captured to encompass the area of interest. Offline, Z-stacks were converted into maximum projections and stitched together with ImageJ (*63*). Counting of tdTomato- and mCherry-labeled cells was performed using ImageJ. To count cells in ImageJ, first, the ROI was isolated and converted to a grayscale image. Background was subtracted, and a binary image was created using rolling ball. To separate overlapping objects, the binary image underwent watershed separation. Last, the Analyze Particles plugin was run, and the minimum particle size was adjusted to exclude objects that were too small to be cell somata. For experiments including mCherry nuclear labeling, mCherry labeling was only counted if it was associated with DAPI nuclear labeling. For AC labeling experiments (Fig. 1, C to F), five slices were used per brain, with ~300 um in between each used slice. For MGB labeling experiments (Fig. 3, A to D, and fig. S3), 10 slices were used per brain, with ~50 um in between each used slice. In addition to cell bodies, some diffuse and punctate staining was observed in the AC of these mice (Fig. 1C), particularly in areas with a high density of labeled cells. This fluorescent signal is likely the result of labeled axons and dendrites from tdTomato-positive neurons. Brain areas were determined using Allen Mouse Brain Atlas (*64*) ( 2011 Allen Institute for Brain Science, Allen Mouse Brain Atlas; http://mouse.brain-map.org/static/atlas) and structural features. Images with MGB subdivisions (Fig. 3 and fig. S3) were generated using vector maps from KimLab Unified Anatomical Atlas (https://kimlab.io/brain-map/atlas/) (*65*), which were adjusted to fit histological landmarks and anti-calbindin staining, as calbindin is labeled primarily only in the dorsal and medial portions of the MGB (*37*).

### Slice electrophysiology

Slice electrophysiology experiments were performed in mice, at least 12 days (P40 to P47 in AC) or 30 days (P58 to P65 in MGB) after viral vector and colored microspheres injections.

Mice were first anesthetized with isoflurane and then immediately decapitated. Brains were rapidly removed, and coronal slices (300 μm) containing the right AC were prepared in a cutting solution at 1°C using a Vibratome (VT1200 S, Leica). The cutting solution, pH 7.4, ∼300 mOsm, contained the following: 2.5 mM KCl, 1.25 mM NaH_2_PO_4_, 25 mM NaHCO_3_, 0.5 mM CaCl_2_, 7 mM MgCl_2_, 7 mM glucose, 205 mM sucrose, 1.3 mM ascorbic acid, and 3 mM sodium pyruvate (bubbled with 95% O_2_/5% CO_2_). The slices were immediately transferred and incubated at 34°C in a holding chamber for 40 min before recording. The holding chamber contained ACSF, pH 7.4, ∼300 mOsm, containing the following: 125 mM NaCl, 2.5 mM KCl, 26.25 mM NaHCO_3_, 2 mM CaCl_2_, 1 mM MgCl_2_, 10 mM glucose, 1.3 mM ascorbic acid, and 3 mM sodium pyruvate (pH 7.4, ∼300 mOsm) (bubbled with 95% O_2_/5% CO_2_). Contaminating zinc was removed from the ACSF by stirring the ACSF with Chelex 100 resin (Bio-Rad) for 1 hour (*8*). After the filtering of ACSF from Chelex resin, using Nalgene rapid flow filters lined with polyethersulfone (0.2-μm pore size), high-purity MgCl_2_·6H_2_O and CaCl_2_·2H_2_O salts (99.995% purity; Sigma-Aldrich) were added. All plastic and glassware were washed with 5% high-purity nitric acid (Sigma-Aldrich). After incubation, the slices were stored at room temperature until the time of recording. Whole-cell recordings in voltage-clamp mode were performed on slices bathed in carbogenated ACSF, which was identical to the incubating solution. Borosilicate pipettes (World Precision Instruments) were pulled into patch electrodes with 2.5- to 5-megohm resistance (Sutter Instruments) and filled with a cesium-based internal solution containing 126 mM CsCH_3_O_3_S, 4 mM MgCl_2_ 10 mM Hepes, 4 mM Na_2_-adenosine 5′-triphosphate (ATP), 0.3 mM tris–guanosine 5′-triphosphate (GTP), 10 mM tris-phosphocreatine, 1 mM CsEGTA, 1 mM QX-314, and 3 mM sodium ascorbate (pH 7.25, 295 mOsm), which was used for recording NMDA_A_R EPSCs in whole-cell voltage clamp. For recording AMPA_R_ EPSCs, a potassium-based intracellular solution containing 128 mM K-gluconate, 10 mM Hepes, 4 mM MgCl_2_, 4 mM Na_2_-ATP, 0.3 mM tris-GTP, 10 mM tris-phosphocreatine, 1 mM EGTA, and 3 mM sodium ascorbate (pH = 7.25, 295 mOsm) was used.

Electrophysiological recordings were made using a MultiClamp-700B amplifier equipped with Digidata-1440A A/D converter and Clampex (Molecular Devices). Data were sampled at 10 kHz and filtered at 3 kHz. Pipette capacitance was compensated and series resistance for recordings was lower than 15 megohms. Series resistance (*R*_series_) was determined by giving a −5-mV voltage step for 50 ms in voltage-clamp mode (command potential set either at −70 mV or at +40 mV) and was monitored throughout the experiments. *R*_series_ was calculated by dividing the −5-mV voltage step by the peak current value generated immediately after the step in the command potential. Input resistance (*R*_input_) was calculated by giving a −5-mV step in voltage-clamp mode (command potential set either at −70 mV or at +40 mV), which resulted in transient current responses. The difference between baseline and steady-state hyperpolarized current (Δ*I*) was used to calculate *R*_input_ using the following formula: *R*_input_ = −5 mV/Δ*I* − *R*_series_. Recordings were excluded from further analysis if the series or input resistance changed by more than 15% compared to the baseline period. In vitro and in vivo electrophysiology experiments with ZnT3-CreERT2^+/+^/Rox^+/+^-mCherry, ZnT3-CreERT2^−/−^/Rox^−/−^-mCherry, and ZnT3 KO mice were performed with genotype blinded to the experimenter.

### Light-evoked excitatory postsynaptic currents

Lev-EPSCs were evoked by optogenetic stimulation of presynaptic axons. More specifically, a collimated blue LED light source (470 nm; Thorlabs) was directed through a diaphragm and a 40× microscope objective and restricted to a small spot adjacent to the recording neuron. The minimal light intensity to elicit a reliable response was determined on a cell-by-cell basis with 0.15-ms duration remaining constant. Trials included a single pulse with an intertrial interval of 30 s. NMDAR responses (NMDAR EPSCs) were recorded in voltage-clamp mode at +40 mV (peak values were averaged over a 20-ms time window) in the presence of 6,7-dinitroquinoxaline-2,3-dione (DNQX) (10 μM; AMPAR and kainate receptor antagonist) and SR 95531 hydrobromide (Gabazine) (20 μM; GABA_A_R antagonist). For NMDAR responses, we applied tetrodotoxin (1 μM) to isolate monosynaptic excitatory inputs. AMPAR responses (AMPAR EPSCs) were recorded in voltage-clamp mode at −70 mV (peak values were averaged over a 1-ms time window). To investigate the effect of ZX1, AMPAR and NMDAR EPSCs were recorded under baseline conditions for at least 5 min and for 20 min following 100 μM ZX1 application. ZX1 takes approximately 15 min to diffuse properly throughout the AC and thus reaches its maximum effect after 15 min of application (*8*, *9*). Representative traces for Figs. 4 and 6 and fig. S4 were acquired by averaging 10 consecutive Lev-EPSCs.

### Statistical analysis

For data that passed the Shapiro-Wilk normality test, paired *t* tests were used for statistical analyses to compare the effect of drug application on responses in the absence of other statistical factors (for example, genotype). Otherwise, we used the Wilcoxon signed-rank test for non-normally distributed data. For the statistical comparisons between two independent groups that passed the Shapiro-Wilk normality test, we used unpaired *t* tests. Otherwise, we used the Mann-Whitney rank-sum test for non-normally distributed data. For comparisons between multiple factors, such as mouse genotype and drug effects, we assessed overall differences with a two-way analysis of variance (ANOVA) followed by pairwise comparisons using the Holm- Bonferroni correction for multiple comparisons. For in vivo imaging experiments, a permutation test was used for two sample comparisons. Samples for which 5000 of 100,000 random permutations of the data resulted in mean differences greater than the observed difference in sample means were considered significant. Significance levels are denoted as *P < 0.05, **P < 0.01, and ***P < 0.001. The details of statistical tests are described in the figure legends and table S1. Group data are presented as means ± SEM. In addition, when we refer *n* = X cells from Y mice, then “*n*” corresponds to the numbers of cells.

## Notes

View/request a protocol for this paper from *Bio-protocol*.
