# Supplementary material for: A CRE/DRE dual recombinase transgenic mouse reveals synaptic zinc–mediated thalamocortical neuromodulation
Source: Sci Adv. 2023 Jun 9;9(23):eadf3525. doi: 10.1126/sciadv.adf3525 (PMC10256168; doi:10.1126/sciadv.adf3525)
Supplement: Supplementary file 1 — Figs. S1 to S4 Table S1 [file sciadv.adf3525_sm.pdf]

Science Advances
AAAS

Supplementary Materials for

A CRE/DRE dual recombinase transgenic mouse reveals synaptic zinc–mediated thalamocortical neuromodulation

Stylianos Kouvaros *et al.*

Corresponding author: Thanos Tzounopoulos, thanos@pitt.edu; Stylianos Kouvaros, stkouvaros@gmail.com; Michael Michaelides, mike.michaelides@nih.gov

Sci. Adv. **9**, eadf3525 (2023)
DOI: 10.1126/sciadv.adf3525

This PDF file includes:

Figs. S1 to S4
Table S1

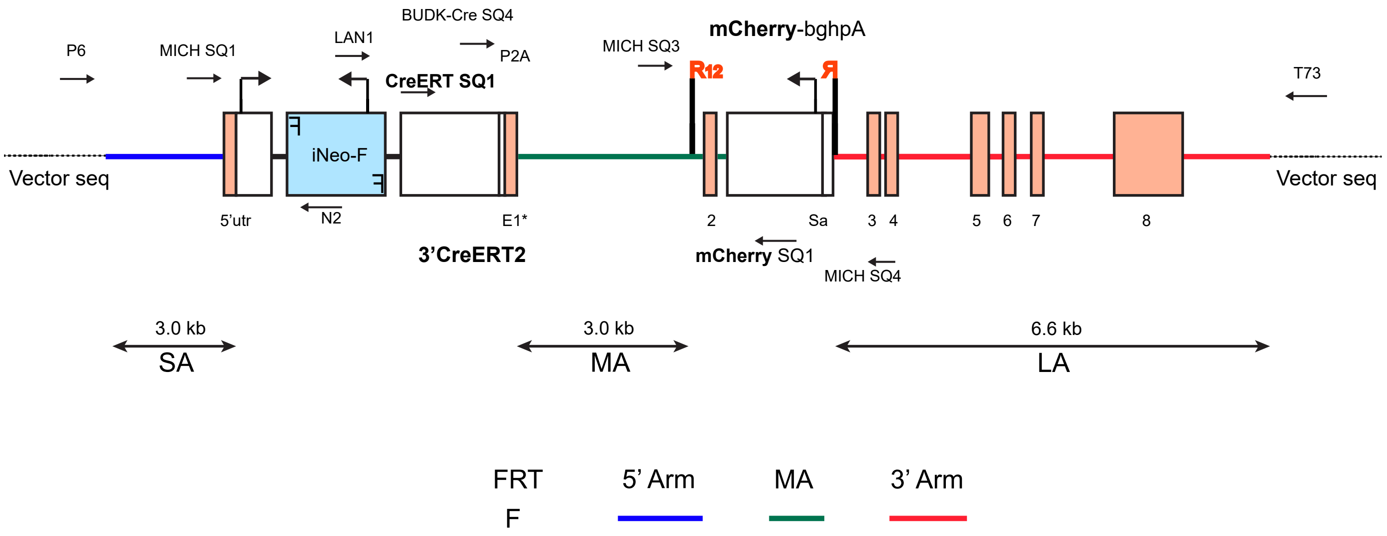

Fig. S1. A schematic diagram of the targeting vector. A 13 kb genomic DNA used to construct the targeting vector was first subcloned from a positively identified C57BL/6 BAC clone. The region was designed such that the 3' long homology arm (LA) extends ~6.6 kb 3' to the Rox12/Rox flanked mCherry inversion cassette, and the 5' short homology arm (SA) extends about 3 kb 5' to the 5' Cre sequence. The 5' Cre is in-frame fused to the endogenous ATG start site. The 3' CreERT2 is fused with P2A-exon 1 coding sequence. The mCherry inversion cassette is inserted in intron 2. Exon 2 and the inversion cassette are flanked by ROX12/ROX sites (red 'R') for conditionally expressing mCherry reporter.

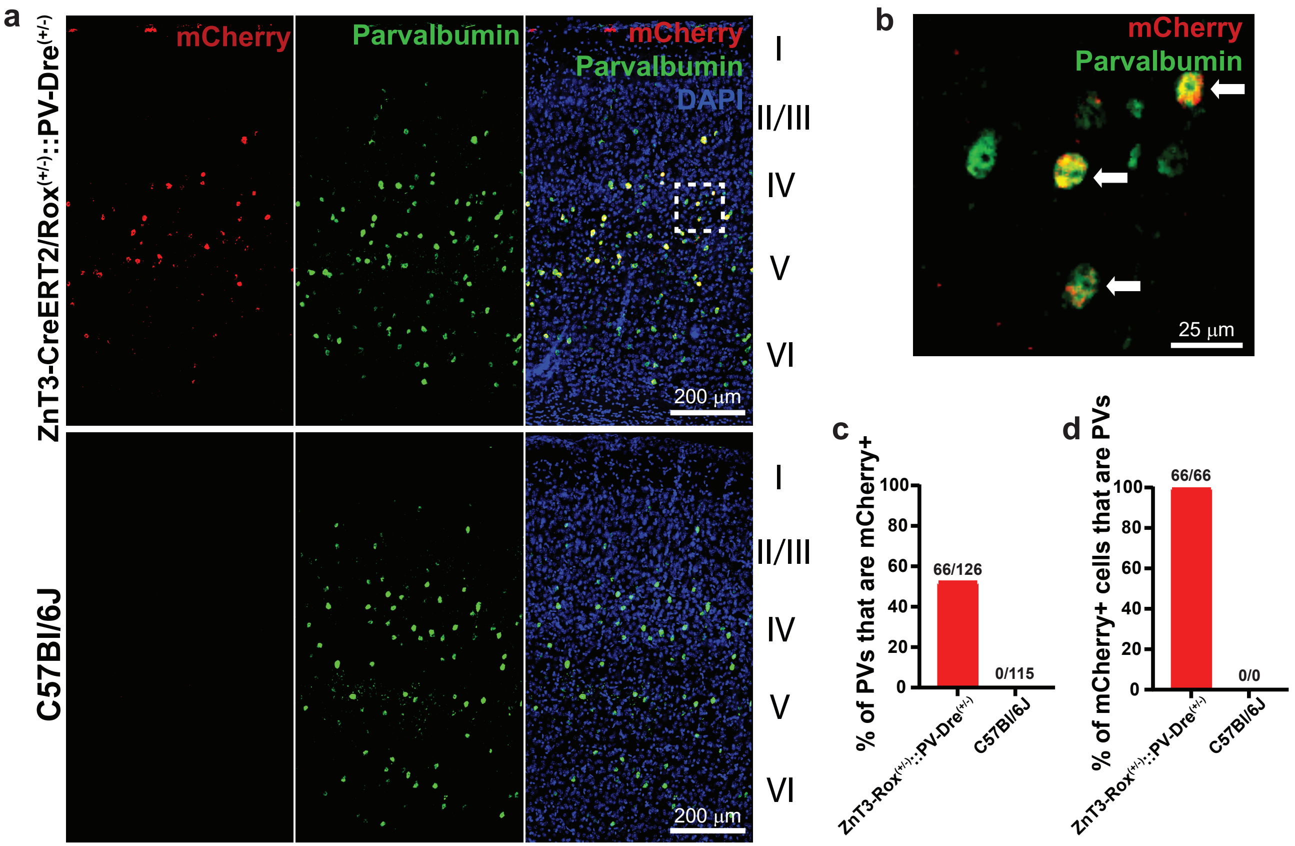

Fig. S2. Cell-type-specific DreO-mediated recombination in PV neurons. a) Representative images of ZnT3-CreERT2$^{+/-}$/Rox$^{+/-}$-mCherry::PV-Dre$^{+/-}$ (top) and C57Bl/6J mice (bottom) brain slices labeled with anti-mCherry (red) and anti-PV (green) antibodies, as well as DAPI (blue). Yellow cells indicate co-labeling for mCherry and PV. **b)** High magnification image of area indicated by white box in **(a)**. White arrows indicate neurons co-labeled for PV and mCherry. **c)** Quantification of the percentage of PV neurons that are co-labeled for mCherry. **d)** Quantification of the percentage of mCherry-labeled cells that are co-labeled for PV.

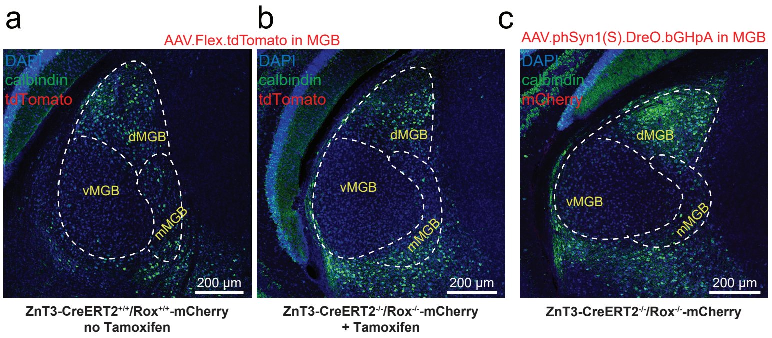

Fig. S3: Control MGB experiments for the CRE-ERT2 and Rox loci. a) Representative MGB image of ZnT3-CreERT2$^{+/+}$/Rox$^{+/+}$ mice not treated with tamoxifen. **b)** Representative MGB image of ZnT3-CreERT2$^{-/-}$/Rox$^{-/-}$ mice treated with tamoxifen. No tdTomato labeled neurons were observed (n=3 mice per group/10 slices per mouse). **c)** Representative MGB image in ZnT3-CreERT2$^{-/-}$/Rox$^{-/-}$ mice injected with AAV.phSyn1(s).DreO.bGHpA. No mCherry labeled cells were observed (n=3 mice per group/10 slices per mouse). Anti-calbindin labeling (green) was used to determine MGB subregions.

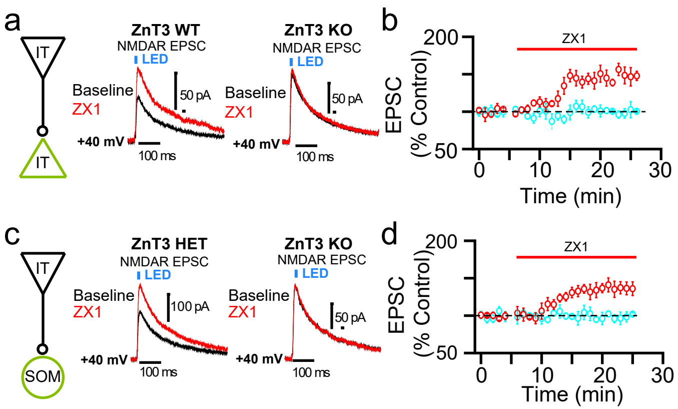

Fig. S4: ZnT3-dependent vesicular zinc inhibits NMDAR EPSCs in AC L2/3 PNs and SOMs.
a-b) Zincergic synapses between IT pyramidal neurons in AC L2/3. **a)** Left: Synapse illustration. Middle: Representative traces of PN NMDAR Lev-EPSCs in baseline (black) and after ZX1 (red) in ZnT3 WT mice. Right: Same as the middle panel but in ZnT3 KO mice. **b)** Time course of the average amplitude of PN NMDAR Lev-EPSCs before and after ZX1 in ZnT3 WT (red) and ZnT3 KO (light blue) mice ($p < 0.0001$, $n = 5$ cells from 3 ZnT3 WT mice, and $n = 5$ cells from 4 ZnT3 KO mice). Error bars indicate ± SEM. **c-d)** Zincergic synapses between IT pyramidal neurons and SOM interneurons. **c)** Left: Synapse illustration. Middle: Representative traces of SOM NMDAR Lev-EPSCs in baseline (black) and after ZX1 (red) in SOM-GFP / ZnT3 Het mice. Right: Same as the left panel but in SOM-GFP / ZnT3 KO mice. **d)** Time course of the average amplitude of SOM NMDAR Lev-EPSCs before and after ZX1 in SOM-GFP / ZnT3 Het (red) and SOM-GFP / ZnT3 KO (light blue) mice ($p < 0.0001$, $n = 5$ cells from 5 ZnT3 HET mice, and $n = 4$ cells from 4 ZnT3 KO mice). Error bars indicate ± SEM.

Figure	Comparison	Statistical Test	F, p or p, t, df	N (cells and mice)
**2d-e**	ZnT3-CreERT2$^{-/-}$ / Rox$^{-/-}$ control<br>vs<br>ZnT3-CreERT2$^{+/+}$/Rox$^{+/+}$ control	*Mixed-effects model*<br>Main effect of ZnT3<br>Interaction of Sound intensity x ZnT3	F (1, 9) = 3.5197<br>F (10, 90) = 0.6371	ZnT3-CreERT2-/- / Rox-/-: 5 mice<br><br>ZnT3-CreERT2$^{+/+}$/Rox$^{+/+}$: 6 mice
**4h**	ZnT3 Control (tdTomato) vs ZnT3 KO (DreO)	*2-way ANOVA*<br>Effect of ZnT3 KO<br><br>Effect of ZX1<br><br>ZX1 x ZnT3 KO interaction	F (1, 250) = 122.7<br>p < 0.0001<br>F (24, 250) = 5.437<br>p < 0.0001<br>F (24, 250) = 6.000<br>p < 0.0001	Control: 6 cells from 3 mice<br><br>ZnT3 KO: 6 cells from 3 mice
**6b**	IT→IT NMDAR EPSCs<br>Baseline vs ZX1	*Paired t-Test*<br>Effect of ZX1	p = 0.007<br>t = -3.999<br>df = 6	7 cells from 5 mice
**6d**	IT→IT AMPAR EPSCs<br>Baseline vs ZX1	*Wilcoxon sign rank test*<br>Effect of ZX1	p = 0.237<br>t = -1.183<br>df = 6	7 cells from 4 mice
**6f**	IT→SOM NMDAR EPSCs<br>Baseline vs ZX1	*Paired t-Test*<br>Effect of ZX1	p = 0.003<br>t = -5.258<br>df = 5	6 cells from 6 mice
**6h**	IT→SOM AMPAR EPSCs<br>Baseline vs ZX1	*Paired t-Test*<br>Effect of ZX1	p = 0.011<br>t = 3.212<br>df = 9	10 cells from 10 mice
**6j**	IT→PV NMDAR EPSCs<br>Baseline vs ZX1	*Paired t-Test*<br>Effect of ZX1	p = 0.015<br>t = -4.052<br>df = 4	5 cells from 5 mice
**6l**	IT→PV AMPAR EPSCs<br>Baseline vs ZX1	*Paired t-Test*<br>Effect of ZX1	p = 0.388<br>t = 1.096<br>df = 3	4 cells from 4 mice
**S4b**	IT→IT NMDAR EPSCs<br>ZnT3 WT vs ZnT3 KO	*2-way ANOVA*<br>Effect of ZnT3 KO<br><br>Effect of ZX1<br><br>ZX1 x ZnT3 KO interaction	F (1, 150) = 160.5<br>p < 0.0001<br>F (24, 150) = 5.558<br>p < 0.0001<br>F (24, 150) = 4.811<br>p < 0.0001	ZnT3 WT: 5 cells from 3 mice<br><br>ZnT3 KO: 5 cells from 4 mice
**S4d**	IT→SOM NMDAR EPSCs<br>ZnT3 HET vs ZnT3 KO	*2-way ANOVA*<br>Effect of ZnT3 KO<br><br>Effect of ZX1<br><br>ZX1 x ZnT3 KO interaction	F (1, 175) = 85.87<br>p < 0.0001<br>F (24, 175) = 2.799<br>p < 0.0001<br>F (24, 175) = 3.269<br>p < 0.0001	ZnT3 HET: 5 cells from 5 mice<br><br>ZnT3 KO: 4 cells from 4 mice

Table S1. Statistical details for experiments in Figs. 2, 4, 6 and S4
